# Serum S100B: A proxy marker for grey and white matter status in the absence and presence of (increased risk of) psychotic disorder?

**DOI:** 10.1371/journal.pone.0174752

**Published:** 2017-03-30

**Authors:** Christine van der Leeuw, Sanne Peeters, Ed Gronenschild, Stijn Michielse, Marcel Verbeek, Paul Menheere, Jim van Os, Machteld Marcelis, on behalf of Genetic Risk and Outcome in Psychosis (G.R.O.U.P.)

**Affiliations:** 1 Department of Psychiatry and Neuropsychology, School for Mental Health and Neuroscience, EURON, Maastricht University Medical Centre, Maastricht, the Netherlands; 2 Faculty of Psychology and Educational Sciences, Open University of the Netherlands, Heerlen, the Netherlands; 3 Departments of Neurology and Laboratory Medicine, Radboud University Medical Centre, Nijmegen, the Netherlands; 4 Donders Institute for Brain, Cognition and Behaviour, Nijmegen, the Netherlands; 5 Central Diagnostic Laboratory, Maastricht University Medical Centre, Maastricht, the Netherlands; 6 Department of Psychosis Studies, Institute of Psychiatry, King’s College London, United Kingdom; 7 Institute for Mental Health Care Eindhoven (GGzE), Eindhoven, the Netherlands; United (Osaka U, Kanazawa U, Hamamatsu U Sch Med, Chiba U and Fukui U) Graduate School of Child Developmen, JAPAN

## Abstract

S100B is a protein with dose-dependent neurotrophic and neurotoxic effects. Whether S100B in psychotic disorder mirrors pathophysiological mechanisms (which elicit exacerbation of disease) or compensatory action is unclear, as is its validity as a proxy marker for brain status. Insight may be gained by examining associations between serum S100B and indices of grey (cortical thickness (CT)) and white matter (fractional anisotropy (FA)), in relation to the absence or presence of (increased risk of) psychotic disorder. Blood samples and cerebral magnetic resonance imaging (MRI) scans were acquired in 32 patients with psychotic disorder, 44 non-psychotic siblings of patients with psychotic disorder and 26 controls. Interactions between S100B and group were examined in separate models of CT and FA measures with multilevel regression analyses weighted for number of vertices and voxels (i.e. units of volume) respectively. All analyses were adjusted for sex, age, body mass index (BMI), scan sequence, handedness and highest level of education. Neither CT nor FA was associated with S100B. There were no significant S100B × group interactions (CT: χ^2^ = 0.044, p = 0.978; FA: χ2 = 3.672, p = 0.159). No evidence was present for S100B as a proxy marker of grey or white matter status. The association between S100B and brain measures was not moderated by psychosis risk.

## Introduction

S100B is a protein with dose-dependent neurotrophic and neurotoxic effects. Previously, we investigated serum protein S100B as a marker of familial risk of psychotic disorder. We did not find S100B elevation in unaffected siblings of patients with psychotic disorder. Nor was S100B elevated in patients with psychotic disorder in our samples (including patients generally not in an acute stage of the disorder, with a mean illness duration <10 years) [1]. The absence of S100B elevation in patients and siblings in our study, in combination with the previously reported positive findings in predominantly hospitalized patients (with more severe psychopathology), suggests that S100B elevations may reflect non-remitted illness processes in certain patient populations. A systematic review and meta-analysis [2], which included our study, showed a significant increase in S100B in patients with schizophrenia which remained present after subgroup analyses to account for clinical and methodological heterogeneity (although heterogeneity remained high in the subgroup analyses). Whether S100B in psychotic disorder mirrors pathophysiological mechanisms (which elicit exacerbation of disease) or compensatory action is unclear.

Aleksovska and colleagues [2] suggest that S100B is a candidate biomarker of neuroplasticity although there is little literature to illustrate this. One study associated S100B overexpression in transgenic mice with neurogenesis in the hippocampus in stressful conditions [3]. In other animal studies, posttraumatic intraventricular infusion of S100B has been shown to benefit hippocampal neurogenesis and cognitive function [4, 5]. Human studies are at an earlier stage. Streitbürger and colleagues [6] were the first to test the validity of S100B as a peripheral marker for the human brain. Their study demonstrated that S100B was negatively correlated with fractional anisotropy (FA) and positively correlated with radial diffusivity in healthy females, possibly signaling diminished fiber directionality and demyelination. No associations between S100B and grey matter indices were found.

The current study may be considered a replication study of Streitbürger’s work [6], confirming or refuting their finding of S100B as a biomarker for the brain in healthy individuals. Furthermore, we wished to investigate whether a similar association exists in patients with a psychotic disorder. To date, the validity of S100B as a proxy marker for white and grey matter status has not been investigated in patients with psychotic disorder or other psychiatric morbidity. We studied the potential association between serum S100B and neuroimaging markers in patients with psychotic disorder, their non-psychotic siblings and controls. Our aim was to assess S100B-associated alterations in brain structure in the context of increased risk (patients and siblings) and disease-effects (patients) pertaining to psychotic disorder. In other words, we tested whether a potential association between S100B and brain measures was moderated by psychosis risk.

Although we previously reported an absence of association between S100B levels and (risk of) psychotic disorder [1], we deemed it warranted to carry out further analyses for two main reasons. First, the meta-analysis by Aleksovska and colleagues, which included our original study, did find elevated S100B blood levels in schizophrenia [2]. Second, the absence of group differences in S100B levels does not rule out an association between S100B and brain measures, that may or may not be moderated by psychosis risk.

## Materials and methods

### Participants

Data was collected in the context of a longitudinal study in the Netherlands [7, 8], specifically in Maastricht and surroundings (including representative parts of Belgium). Patients with a minimum age of 16 years with a diagnosis of non-affective psychosis were included. They were recruited through the mental health services where they were treated, either as outpatients (majority of the sample) or inpatients. Diagnosis was based on DSM-IV criteria (APA, 2000), assessed with the Comprehensive Assessment of Symptoms and History (CASH) interview [9]. Siblings were sampled through participating patients. Control subjects were recruited from the same area as described above, using random mailings in nearby municipalities and through advertisement in newspapers. The CASH was also used to confirm the absence of a diagnosis of non-affective psychosis in the siblings, and absence of lifetime diagnosis of psychotic disorder in the control subjects. For the control subjects, the occurrence of any psychotic disorder in either the subject or any first-degree family member, assessed using the Family Interview for Genetic Studies, constituted an exclusion criterion. Sufficient command of the Dutch language was an additional criterion for inclusion.

Additional exclusion criteria constituted oncologic processes, autoimmune disease, current infectious disease, cardiovascular disease and neurological disease; due to S100B elevation associated with these types of pathology.

The cohort consisted of 32 patients with a psychotic disorder, 44 siblings and 26 controls. Of note, the present study population is a subsample of the Maastricht cohort from our original S100B study [1]. The subsample comprised all participants of whom both blood samples and magnetic resonance imaging (MRI) scans were available. Therefore, this sample is a random result of logistics, i.e. no specific selection was made from the original sample based on demographic characteristics.

Of the patients, 21 were diagnosed with schizophrenia and 9 were diagnosed with schizoaffective disorder. One patient was diagnosed with psychotic disorder not otherwise specified (NOS) and one patient had a diagnosis of substance-induced psychotic disorder. As non-psychotic psychiatric morbidity was not an exclusion criterion for siblings and controls, psychiatric diagnoses were present in these groups. The sample thus included 17 siblings and 5 controls with a history of major depressive disorder (MDD).

A total of 14 families participated in the study. Nine families contributed one patient and one sibling and one family contributed one patient and two siblings. Three families contributed two siblings, but no patients. One family contributed three siblings, but no patients. Twenty-two independent patients, 24 independent siblings and 26 independent controls participated, i.e. these individuals had no family ties within the sample.

### Blood sample acquisition and processing

Blood serum samples were acquired by venipuncture and were centrifuged within 24 hours and frozen until analysis. S100B was analyzed using a Liaison automated chemiluminescence analyzer according to the manufacturer's instructions (Diasorin). The lowest concentration of detection was 0.02 microgram per liter. Receiver operator curves showed best accuracy for the Liaison Sangtec 100 assay [10].

### MRI acquisition and processing

MRI scans were obtained at Maastricht University, the Netherlands, using an Allegra syngo MR A30 (Siemens, Erlangen, Germany) operating at 3.0 Tesla. The following anatomical scan parameters were used: Modified Driven Equilibrium Fourier Transform (MDEFT) sequence; 176 slices, 1 mm isotropic voxel size, echo time 2.4 msec, repetition time 7.92 msec, inversion time 910 msec, flip angle 15°, total acquisition time 12 min 51 sec; Magnetization Prepared Rapid Acquisition Gradient-Echo (MPRAGE; Alzheimer’s Disease Neuroimaging Initiative (ADNI)) sequence 192 slices, 1 mm isotropic voxel size, echo time 2.6 msec, repetition time 2250 msec, inversion time 900 msec, flip angle 9°, total acquisition time 7 min 23 sec. The matrix size was 256 x 256 and field of view was 256 x 256 mm^2^. The number of excitations was one. Due to a scanner update during data collection nine participants (3 patients, 5 siblings and one control) underwent the MDEFT sequence, while for the majority the ADNI sequence was used.

Microstructural white matter anatomy was examined using diffusion tensor imaging (DTI) with an echo-planar-imaging sequence (field of view 230 x 230 mm^2^, TR 10800 ms, TE 84 ms, voxel size 1.8 x 1.8 x 1.8 mm^3^, b-value 1000 s/mm^2^, noise level 40, 85 slices, no overlap). As a result of the scanner update, two DTI sequences were used: one with 76 directions (of which 4 T2-weighted (B0) and 72 diffusion-weighted (B)) and one with 81 directions (8xB0 and 73xB). Only ten participants (3 patients, 6 siblings and 1 control) underwent the sequence with 76 directions. Total acquisition time of the DTI sequence was 15 minutes. Two male siblings did not complete the DTI sequence and were excluded from the analysis.

### Cortical thickness (CT) measurement

Scans were processed and analyzed using Freesurfer stable release v5.3.0, http://surfer.nmr.mgh.harvard.edu [11–15]. To measure CT, the cerebral cortex was parcellated into units based on gyral and sulcal structure [16, 17]. Furthermore, a variety of surface-based data was created including maps of curvature and sulcal depth. This method used both intensity and continuity information from the entire three dimensional MR volume in segmentation and deformation procedures to produce representations of CT, calculated as the closest distance from the grey/white matter boundary to the grey matter/cerebrospinal fluid (CSF) boundary at each vertex on the tessellated surface [12]. The maps were created using spatial intensity gradients across tissue classes and were not restricted to the voxel resolution of the original data, thus were capable of detecting sub-millimeter differences between groups. CT measurement procedures have been validated against histological analysis [18] and manual measurements [19, 20]. Individual CT values for each predefined region of interest (hereafter: ROI; adapted from the Desikan atlas [16], 34 ROIs per hemisphere) were derived by FreeSurfer and exported to R version 3.2.1. Thus, every individual had 68 CT measurements over the predefined ROIs.

### Fractional anisotropy (FA) measurement

Processing of DTI data was effectuated using tract-based spatial statistics (TBSS) v1.2 in FSL 4.1.6 (FMRIB Analysis Group, Oxford, UK, http://www.fmrib.ox.ac.uk/analysis/research/tbss). First, standard Siemens DICOM files were transformed into NIFTI format using a custom built in-house software package named GIANT (General Image ANalysis Tools developed by EHBMG). Raw data were corrected for head movement and eddy currents invoked during scanning. The B0 volume was skull-stripped using FSL's Brain Extraction Tool [21] and this served as a brain mask for all B volumes. The next step was fitting a diffusion tensor model at each voxel using data output from the brain extraction, diffusion weighted data and gradient directions following a general linear model (FreeSurfer v5.3.0). After tensor fitting the process continued working on FA volumes, eroding them slightly.

Nonlinear registration aligned each FA volume to 1 x 1 x 1 mm standard FMRIB58_FA space. The standard FMRIB58_FA contains a template derived from high-resolution images of 58 participants in a well-aligned population (both males and females ranging between 20 and 50 years of age) [22]. After nonlinear transformation of the FA volumes into standard space, a mean FA skeleton from all participants per group was derived. The mean FA skeleton follows the major white matter tracts in each individual participant (normalized in MNI152 space) and provides a way to compare between (groups of) participants. The final step of the processing was setting the FA threshold using visual inspection of the FA skeleton, in the present study at a level of 0.25, to include major white matter tracts whilst removing small peripheral tracts that would cause excess interparticipant variability. In addition, this threshold setting avoided inclusion of regions that are likely to be composed of multiple tissue types or fiber orientations.

The Johns Hopkins University International Consortium for Brain Mapping (JHU ICBM)-DTI-81 white matter atlas labels [23] were used to assign specific tract names. From all 38 JHU labeled white matter tracts, mean FA values were extracted and exported to R version 3.2.1.

### Ethics statement

The study was approved by the standing ethics committee of Maastricht University Medical Centre and all participants gave written informed consent in accordance with the committee’s guidelines. When a participant was younger than 18 years of age, a parent co-signed the informed consent form. Patients were outpatients, generally not in an acute illness stage. They were approached directly, they made independent appointments in order to participate and were supplied with ample verbal and written patient information before they provided informed consent. Thus, in order to participate, their mental competence had to be evident both by goal-directed behaviour enabling participation and from in-person contact when the study was explained and informed consent was obtained. Furthermore, if a sibling was also a participant, patients were frequently accompanied by this sibling.

### Statistical analyses

Conform previous work in this sample [7], datasets were transformed from a wide to long format resulting in a hierarchical structure, with 68 regional CT and 38 DTI measures (level 1) nested in respectively 102 and 100 participants (level 2). Given the clustering of brain measures within participants and participants within families, compromising statistical independence of observations, multilevel random regression models were fitted [24]. R version 3.2.1 was used, employing the nlme, car and multcomp packages. Since outcomes (CT and FA) represent means (based on varying numbers of volumetric units, vertices and voxels respectively, per region), the analyses were weighted based on the number of vertices/voxels per region.

Group × S100B interaction terms were evaluated using Wald-type tests [25]. A priori planned stratified analyses per group were conducted. All analyses were adjusted for sex, age, body mass index (BMI), scan sequence, handedness and highest level of education. In the patient group, additional analyses were conducted correcting for current antipsychotic medication (AP) exposure by converting the daily dose to haloperidol equivalents (in milligrams).

In order to increase power, sensitivity analyses were conducted in healthy individuals (combining controls and siblings) and individuals with (increased risk of) psychotic disorder (combining siblings and patients).

Due to Streitbürger’s sex-specific findings [6], S100B × sex and S100B × group × sex interactions were evaluated in CT and FA models.

## Results

### Descriptive analyses

There was an uneven sex distribution in the patient group, with fewer females (28%) than males. Controls were slightly older than siblings and patients, although not significantly so (Table 1).

**Table 1:** Demographic characteristics.

	Controls	Siblings	Patients
Number of participants	26	44	32
Sex (male/female)	11/15	24/20	25/7
S100B (μg/l)	0.083 ± 0.031	0.087 ± 0.044	0.067 ± 0.029
CT	2.539 ± 0.399	2.546 ± 0.397	2.496 ± 0.395
FA	0.588 ± 0.081	0.575 ± 0.082	0.572 ± 0.085
Age	33.77 ± 11.16	30.39 ± 7.96	30.78 ± 6.49
BMI	23.65 ± 3.36	24.15 ± 4.38	25.99 ± 4.21
Present use of AP (yes/no)			27/5
Haloperidol equivalents (mg)			4.38 ± 3.37

Means ± standard deviations reported.

Patients (P) had a higher mean BMI than controls (C) and siblings (S), the latter at trend-level (P vs. C: B = 2.331, 95% CI 0.188 to 4.475, p = 0.033; P vs. S: 1.835, 95% CI -0.051 to 3.720, p = 0.056), whereas siblings and controls did not differ significantly (S vs. C: B = 0.497, 95% CI -1.511 to 2.505, p = 0.625).

Twenty-seven out of 32 patients reported current use of antipsychotic (AP) medication. Nine patients used risperidone, four used olanzapine, three used aripiprazole, two used clozapine, two quetiapine and two used amisulpride. There was a single case of bromperidol use. One person used a combination of risperidone and paliperidone, one used clozapine and aripiprazole, one person used aripiprazole and olanzapine, and one person used haloperidol and pipamperone.

### Mean S100B, CT and FA

Mean S100B levels appeared lower in patients than in controls and siblings, with siblings having the highest values (see Table 1). Regression analyses corrected for potential confounders (sex, age and BMI) showed that there was no significant difference in S100B serum levels between patients and controls (P vs. C: B = -0.013, 95% CI -0.033 to 0.007, p = 0.208), in line with our first S100B study [1]. However, patients did have lower S100B levels than siblings (P vs. S: B = -0.019, 95% CI -0.036 to -0.002, p = 0.031). There was no significant difference between S100B in siblings and controls (B = 0.006, 95% CI -0.012 to 0.024, p = 0.529).

Mean CT was significantly lower in patients than in controls and siblings (at trend level) when corrected for sex, age, BMI, scan sequence, handedness and highest level of completed education, and weighted for number of vertices (P vs. C: B = -0.049, 95% CI -0.095 to -0.003, p = 0.037; P vs. S: B = -0.036, 95% CI -0.073 to 0.002, p = 0.065), with no significant difference between siblings and controls (B = -0.013, 95% CI -0.054 to 0.027, p = 0.521).

Mean FA was significantly lower in patients and siblings compared to controls, with no significant difference between patients and siblings; again controlled for sex, age, BMI, scan sequence, handedness and highest level of completed education, and weighted for number of voxels (P vs. C: B = -0.016, 95% CI -0.025 to -0.007, p = 0.001; S vs. C: B = -0.013, 95% CI -0.021 to -0.004, p = 0.004; P vs. S: B = -0.003, 95% CI -0.010 to 0.003, p = 0.308).

### Associations between S100B and brain structure

No significant associations between S100B and CT (B = 0.311, 95% CI -0.116 to 0.739, p = 0.154) and S100B and FA (B = 0.030, 95% CI -0.055 to 0.116, p = 0.485) were observed in the total sample.

### Interactions

Neither the group × S100B in the CT model (χ^2^ = 0.044, p = 0.978), nor the group × S100B interaction in the FA model (χ^2^ = 3.672, p = 0.159) were significant, confirmed by stratified analyses (Table 2).

**Table 2:** Associations between S100B and cerebral structural measures.

	Controls	Siblings	Patients
	B (p-value)	B (p-value)	B (p-value)
**Cortical thickness**	0.264 (0.603)	0.287 (0.310)	0.171 (0.720)
**Fractional anisotropy**	-0.135 (0.216)	0.021 (0.702)	0.120 (0.110)

B’s and p-values reported. B represents the regression coefficient of the multilevel regression analyses.

S100B × sex and S100B × group × sex interactions in the CT model were not significant (χ^2^ = 0.135, p = 0.713; and χ^2^ = 2.130, p = 0.345, respectively), nor were those in the FA model (χ^2^ = 0.025, p = 0.874; and χ^2^ = 0.194, p = 0.907, respectively).

### Sensitivity analyses

Combining controls and siblings into one “healthy” group (n = 70) showed no associations between S100B and brain measures (CT: B = 0.274, 95% CI -0.130 to 0.678, p = 0.184; FA: B = -0.053, 95% CI -0.158 to 0.053, p = 0.331). Nor did the combination of siblings and patients into an (at risk of) psychosis group (n = 76) result in significant findings (CT: B = 0.332, 95% CI -0.152 to 0.817, p = 0.179; FA: B = 0.063, 95% CI -0.020 to 0.146, p = 0.136).

Correcting for current AP exposure in the patient group resulted in non-significant associations in both (grey and white matter) models (CT: B = 0.141, 95% CI -0.960 to 1.241, p = 0.802; FA: B = 0.129, 95% CI -0.086 to 0.345, p = 0.240).

## Discussion

We investigated the potential relationship between serum S100B and structural cerebral measures in both healthy subjects (in replication of previous work) and individuals with (increased risk of) psychotic disorder (not examined previously). No significant associations between serum S100B and cerebral structural measures were found in health or in the presence of (increased risk of) psychotic disorder.

### Findings

Our first S100B study showed no association between (risk of) psychotic disorder and S100B [1]. We now report no association between S100B and brain structure in the presence of (increased risk of) psychotic disorder in the same sample. Unfortunately these results cannot be surveyed in the broader context of schizophrenia research as there are no analogous studies (yet) to compare it with.

Furthermore, we do not find that CT and FA are consistently associated with S100B in controls and healthy siblings. The only previous study to explore the association between S100B and brain structure using neuroimaging was performed in healthy controls [6]. This study suggested S100B-related demyelination in women (negative correlation with FA and a positive correlation with radial diffusivity). The analyses conducted by Streitbürger and colleagues were a priori stratified per sex, although they proceed to conclude that true sex-related differential expression patterns of S100B in the brain were not anticipated. The present study explored whether S100B effects on CT and FA were conditional on sex (S100B × sex interaction) and group and sex (S100B × group × sex interaction). No significant interactions were found. Indeed, our original study did not show significant group × sex interactions in two large independent samples [1]. So although sex-specific patterns in neuroimaging studies are well documented, this appears unrelated to levels of S100B in the present study. However, bearing in mind that the sample size is modest the three way interaction likely lacks power.

We did not find any significant associations between S100B and grey matter status represented by cortical thickness in any of the 3 groups. This is in line with Streitbürger’s finding of absence of association between S100B and grey matter density [6] in healthy controls.

Due to the absence of association between serum S100B and brain structure in the current study’s patient group (also after correction for current AP use), we are unable to make any deductions about S100B’s cerebral actions in schizophrenia. S100B has dual effects: at physiological intracellular levels and nanomolar extracellular concentrations it is neurotrophic [26]; at micromolar levels it becomes neurotoxic [26–28]. As Streitbürger [6] and Schmitt [29] have pointed out, S100B elevation in schizophrenia is within the nanomolar range. That is to say that the micromolar S100B concentrations seen in clear neuronal damage (e.g. after traumatic brain injury or infarction) do not seem applicable to schizophrenia. S100B elevation in the nanomolar range is compatible with neurotrophic action. This makes a neurodegenerative process improbable and regenerative action more likely [6, 29].

The interpretation of our results may be complicated by the fact that mean S100B was lowest in patients, significantly so compared to siblings. Noteworthy is the fact that mean S100B was highest in the patient groups in the original study [1], although there were no significant differences in S100B levels between groups in the analyses. As the present sample (a subsample from the original study) is a result of logistics and not of specific selection criteria, we have no explanation for the low S100B levels in the present patient group and therefore conclude that these likely represent chance. Nevertheless, this potentially indicates an issue with power which may result in false negative findings.

### Methodological considerations

There are several limitations in the present study that deserve discussion.

First, scanner updates took place at an early stage during the data collection period. This led to an uneven distribution of scan types (within and between groups). The majority of participants underwent the ADNI sequence and DTI acquisition with 81 directions, though 9 MDEFT sequence scans and 10 DTI scans with 76 directions were obtained. To account for potential confounding by uneven distribution over the groups, scan type was included as a covariate in all analyses. As an extra test, we repeated analyses excluding the MDEFT sequence and the DTI scans with 76 directions. This did not alter the associations between S100B and CT and FA respectively (results not shown).

Second, there were 17 siblings and 5 controls with a history of MDD in this study sample. The findings from our original study indicated that there were no differences in serum S100B between these groups and that the inclusion of MDD and other psychiatric comorbidity in these groups did not alter the pattern of negative findings. As the original study [1] consisted of two independent samples of ample size (allowing for independent replication of our findings) we presume that the findings in controls and siblings were not influenced by MDD. We also base the combination of controls and siblings into a “healthy” group on the premise of absence of associations between S100B and group in the original study.

Third, the patient group was clinically heterogeneous, i.e. inclusion criteria were broader than a diagnosis of schizophrenia. As an extra test, we excluded patients with a diagnosis other than schizophrenia and repeated all analyses (results available upon request). The pattern of findings was not significantly altered.

And finally, mean CT and FA are coarse measures of brain structure. Taking this into consideration, in combination with small sample size, the present study may best be considered as hypothesis-generating.

As this is the first study to investigate an association between S100B and cerebral structural measures in psychotic disorder, no comparison can be made to previous work. Naturally, replication of our study in a larger cohort is required to either corroborate or refute findings.

## Conclusions

No evidence was present for S100B as a proxy marker of grey or white matter status. The association between S100B and brain measures was not moderated by psychosis risk.

## References

[1] van der Leeuw C, Marcelis M, Peeters SC, Verbeek MM, Menheere PP, de Haan L, et al (2013) Replicated Evidence of Absence of Association between Serum S100B and (Risk of) Psychotic Disorder. PLoS One 8: e82535 10.1371/journal.pone.0082535 24358202 PMC3866164

[2] Aleksovska K, Leoncini E, Bonassi S, Cesario A, Boccia S and Frustaci A (2014) Systematic Review and Meta-Analysis of Circulating S100B Blood Levels in Schizophrenia. PLoS One 9: e106342 10.1371/journal.pone.0106342 25202915 PMC4159239

[3] Buschert J, Hohoff C, Touma C, Palme R, Rothermundt M, Arolt V, et al (2013) S100B overexpression increases behavioral and neural plasticity in response to the social environment during adolescence. J Psychiatr Res 47: 1791–1799. 10.1016/j.jpsychires.2013.08.001 23972702

[4] Kleindienst A, McGinn MJ, Harvey HB, Colello RJ, Hamm RJ, Bullock MR. (2005) Enhanced hippocampal neurogenesis by intraventricular S100B infusion is associated with improved cognitive recovery after traumatic brain injury. Journal of neurotrauma 22: 645–655. 10.1089/neu.2005.22.645 15941374

[5] Kleindienst A, Harvey HB, Rice AC, Muller C, Hamm RJ, Gaab MR, et al (2004) Intraventricular infusion of the neurotrophic protein S100B improves cognitive recovery after fluid percussion injury in the rat. Journal of neurotrauma 21: 541–547. 10.1089/089771504774129874 15165362

[6] Streitburger DP, Arelin K, Kratzsch J, Thiery J, Steiner J, Villringer A, et al (2012) Validating Serum S100B and Neuron-Specific Enolase as Biomarkers for the Human Brain—A Combined Serum, Gene Expression and MRI Study. PLoS One 7: e43284 10.1371/journal.pone.0043284 22916238 PMC3422594

[7] Habets P, Marcelis M, Gronenschild E, Drukker M and van Os J (2011) Reduced cortical thickness as an outcome of differential sensitivity to environmental risks in schizophrenia. Biol Psychiatry 69: 487–494. 10.1016/j.biopsych.2010.08.010 20951979

[8] Korver N, Quee PJ, Boos HB, Simons CJ and de Haan L (2012) Genetic Risk and Outcome of Psychosis (GROUP), a multi site longitudinal cohort study focused on gene-environment interaction: objectives, sample characteristics, recruitment and assessment methods. Int J Methods Psychiatr Res 21: 205–221. 10.1002/mpr.1352 22419500 PMC6878383

[9] Andreasen NC, Flaum M and Arndt S (1992) The Comprehensive Assessment of Symptoms and History (CASH). An instrument for assessing diagnosis and psychopathology. Arch Gen Psychiatry 49: 615–623. 1637251 10.1001/archpsyc.1992.01820080023004

[10] Smit LH, Korse CM and Bonfrer JM (2005) Comparison of four different assays for determination of serum S-100B. Int J Biol Markers 20: 34–42. 15832771 10.1177/172460080502000106

[11] Dale AM, Fischl B and Sereno MI (1999) Cortical surface-based analysis. I. Segmentation and surface reconstruction. Neuroimage 9: 179–194. 10.1006/nimg.1998.0395 9931268

[12] Fischl B and Dale AM (2000) Measuring the thickness of the human cerebral cortex from magnetic resonance images. Proc Natl Acad Sci U S A 97: 11050–11055. 10.1073/pnas.200033797 10984517 PMC27146

[13] Fischl B, Salat DH, Busa E, Albert M, Dieterich M, et al (2002) Whole brain segmentation: automated labeling of neuroanatomical structures in the human brain. Neuron 33: 341–355. 11832223 10.1016/s0896-6273(02)00569-x

[14] Fischl B, Sereno MI and Dale AM (1999) Cortical surface-based analysis. II: Inflation, flattening, and a surface-based coordinate system. Neuroimage 9: 195–207. 10.1006/nimg.1998.0396 9931269

[15] Segonne F, Dale AM, Busa E, Glessner M, Salat D, van der Kouwe A, et al (2004) A hybrid approach to the skull stripping problem in MRI. Neuroimage 22: 1060–1075. 10.1016/j.neuroimage.2004.03.032 15219578

[16] Desikan RS, Segonne F, Fischl B, Quinn BT, Dickerson BC, Blacker D, et al (2006) An automated labeling system for subdividing the human cerebral cortex on MRI scans into gyral based regions of interest. Neuroimage 31: 968–980. 10.1016/j.neuroimage.2006.01.021 16530430

[17] Fischl B, van der Kouwe A, Destrieux C, Halgren E, Segonne F, Salat DH, et al (2004) Automatically parcellating the human cerebral cortex. Cereb Cortex 14: 11–22. 14654453 10.1093/cercor/bhg087

[18] Rosas HD, Liu AK, Hersch S, Glessner M, Ferrante RJ, Salat DH, et al (2002) Regional and progressive thinning of the cortical ribbon in Huntington's disease. Neurology 58: 695–701. 11889230 10.1212/wnl.58.5.695

[19] Kuperberg GR, Broome MR, McGuire PK, David AS, Eddy M, Ozawa F, et al (2003) Regionally localized thinning of the cerebral cortex in schizophrenia. Arch Gen Psychiatry 60: 878–888. 10.1001/archpsyc.60.9.878 12963669

[20] Salat DH, Buckner RL, Snyder AZ, Greve DN, Desikan RS, Busa E, et al (2004) Thinning of the cerebral cortex in aging. Cereb Cortex 14: 721–730. 10.1093/cercor/bhh032 15054051

[21] Smith SM (2002) Fast robust automated brain extraction. Hum Brain Mapp 17: 143–155. 10.1002/hbm.10062 12391568 PMC6871816

[22] Smith SM, Jenkinson M, Johansen-Berg H, Rueckert D, Nichols TE, Mackay CE, et al (2006) Tract-based spatial statistics: voxelwise analysis of multi-subject diffusion data. Neuroimage 31: 1487–1505. 10.1016/j.neuroimage.2006.02.024 16624579

[23] Mori S, Oishi K, Jiang H, Jiang L, Li X, Akhter K, et al (2008) Stereotaxic white matter atlas based on diffusion tensor imaging in an ICBM template. Neuroimage 40: 570–582. 10.1016/j.neuroimage.2007.12.035 18255316 PMC2478641

[24] Goldstein H (1987) Multilevel Models in Educational and Social Research. London: Griffin.

[25] Clayton D and Hills M (1993) Statistical Models in Epidemiology. Oxford: Oxford University Press.

[26] Donato R, Cannon BR, Sorci G, Riuzzi F, Hsu K, Weber DJ, et al (2013) Functions of S100 proteins. Curr Mol Med 13: 24–57. 22834835 PMC3707951

[27] Sen J and Belli A (2007) S100B in neuropathologic states: the CRP of the brain? J Neurosci Res 85: 1373–1380. 10.1002/jnr.21211 17348038

[28] van Beveren NJ, van der Spelt JJ, de Haan L and Fekkes D (2006) Schizophrenia-associated neural growth factors in peripheral blood. A review. Eur Neuropsychopharmacol 16: 469–480. 10.1016/j.euroneuro.2006.02.001 16545550

[29] Schmitt A, Bertsch T, Henning U, Tost H, Klimke A, Henn FA, et al (2005) Increased serum S100B in elderly, chronic schizophrenic patients: negative correlation with deficit symptoms. Schizophr Res 80: 305–313. 10.1016/j.schres.2005.04.013 15964742

